# Cardiac Arrest 2012

**DOI:** 10.1186/1757-7241-21-S1-A5

**Published:** 2013-05-28

**Authors:** T Tom Webster, S Sarah Eshelby, AE Anne Weaver

**Affiliations:** 1 Barts and the London Medical School, London, UK; 2 London’s Air Ambulance, London, UK

## 

The 2^nd^ London Cardiac Arrest Symposium provided cardiac professionals the opportunity to discuss the current management of cardiac arrest and key questions regarding this time critical medical emergency. The presentations kindly provided by Zoll ™ can be found online at http://www.londoncardiacarrestsymposium.com/videos-2012.html

Mr Jerry Overton, Chair of the International Academies of Emergency Dispatch opened with discussion of the rationale for the widespread introduction of automated (mechanical) CPR in ambulance services. Only 1-8% of adults suffering a pre-hospital cardiac arrest survive to hospital discharge. Effective CPR is required to perfuse the heart and brain, to re-establish output and achieve survival. Studies have shown that manual CPR by trained professionals achieves 25-30% of normal blood flow. After 3 minutes of manual CPR most rescuers show fatigue and a reduction in the quality of compressions. Studies have demonstrated that effective CPR during transport is virtually impossible. The use of mechanical CPR is this situation could potentially double survival rates. Mr. Overton spoke about the results of three studies-comparing manual and automated CPR. Survival to discharge was 2.9% with manual and 9.7% with mechanical CPR. The addition of simultaneous may deliver further survival advantage.

There were three presentations which concentrated on cardiac arrests due in the young of non-atherosclerotic origin. Dr Andrew Grace a Consultant Cardiologist from Cambridge, focused on cardiac arrest in the young. Dr Anna Green a histopathologist presented on ‘The histopathologist’s view of Sudden Cardiac Death and Professor Sanjay Sharma from St George’s NHS Trust and Medical Director of the London Marathon discussing cardiac arrest in young athletes. Dr Grace focused particularly on the differences in Sudden Arrhythmic Death syndrome (SADs) and Acute Coronary Syndromes. 14% of cardiac deaths are due to SADs, equating to 70,000-100,000 SADs deaths per year. The majority of cases have an underlying genetic cause affecting the sarcomeres, leading to disorganization of the myocardium and predisposition to ventricular fibrillation. Professor Sharma explained that 40% of athlete deaths are under the age of 18 and of these 90% arrest during or within 30 minutes of completing physical exercise. Most worryingly, arrests in young, healthy individuals often occur without prodromal symptoms. Often arrests in young athletes are witnessed and captured on film and have a major media impact. Dr Green emphasized the importance of suspicion when presented with apparently ‘normal hearts’ on macroscopic examination. Further tests often reveal undetected abnormalities. She emphasised the importance of routine molecular autopsy in unexplained cardiac arrest to establish cause and allow family counselling. She also touched on the difficulties of retaining human tissue and the need for standard operating procedures with early involvement of the Coronial system and families. Both Dr Grace and Professor Sharma talked about screening for sudden cardiac death. ECG diagnosis is not always easy. One study showed that even cardiologists struggle to identify a prolonged ECG QT interval in 25% cases. Professor Sharma considered the Italian screening programme for young athletes comprising of history, examination and 12 lead electrocardiogram. Although this has led to a 90% decrease in sudden cardiac death in athletes questions have been raised about cost benefit, specificity and the generation of false negatives. Dr Grace similarly stated that genetic screening will incur significant cost and very high costs per life saved.

A CIRC (Circulation Improving Resuscitation Care) update by Dr. Lars Wik from Oslo University Hospital, concluded the first session. This trial compared the use of the mechanical CPR with excellent quality manual CPR. Dr Wik reported on data from this multi-centre international randomized controlled trial. More than 500 ZOLL “AutoPulse” units were deployed and more than 5,000 medics were trained on the device. Medical professionals enrolled in the study carried out extensive training on the automated CPR device and were also continually evaluated on their manual CPR techniques. Patients were randomly allocated to receive manual or mechanical CPR. Dr Wik suggested that the results will demonstrate that “AutoPulse” is able to achieve a CPR fraction of 80% and outcome improvements.

Professor Simon Redwood, from St. Thomas’ Hospital, London, presented on cardiac interventions during and after cardiac arrest and in particular, the effect of immediate percutaneous coronary intervention (PCI). He spoke about the potential survival advantages of combining early PCI and therapeutic hypothermia and the possibility of using mechanical CPR as a bridge to PCI. He presented the various international guidelines for post-arrest PCI. He believed that any patient who has ROSC following cardiac arrest (of cardiac origin) should undergo PCI regardless of presenting rhythm or ECG findings. He closed his session by highlighting a promising Japanese study combining ECMO and PCI.

The keynote address was given by Professor Maaret Castren, from Stockholm and current Chair of the ERC. Her presentation was entitled “Cool to be Cold” and covered aspects of induced hypothermia as a key intervention in cardiac resuscitation. She highlighted the lack of extensive research in induced hypothermia in cardiac arrest management but advocated a common sense approach. A strategic approach of scientific understanding, education and local implementation is key to improving survivability. In addition to discussing the effectiveness of various methods of cooling, Professor Castren considered the problems of post arrest pyrexia, methods of measuring core temperature and the speed / timing at which cooling should take place. She then highlighted recent advances in cooling and the development of the Rhinochill™ device. A feasibility study of pre-hospital Rhinochill use demonstrated a significant reduction in time to brain cooling. The study also highlighted a significant increase in survivability for patients receiving CPR within 10 minutes of arrest and cooled with Rhinochill (59%) vs patients not cooled with Rhinochill (29%). Professor Castren concluded by drawing on her personal experience and stating that post cardiac arrest patients should be cooled aggressively and have hypothermia maintained effectively. All treatment should be meticulously documented to allow audit.

Dr Jim Connolly from Newcastle, spoke on the use of ultrasound in cardiac arrest. He emphasized the limitations of pulse checks, oximetry and stethoscope use in the initial assessment of cardiac arrest ^9^. He demonstrated how the addition of ultrasound to an algorithm could refine management by early exclusion of hypovolaemia, thrombus, cardiac tamponade and tension pneumothorax.The scarcity of evidence linking ultrasound use to survivability, coupled with specific training requirements appear to have limited the utility of ultrasound on a wide scale, especially in pre-hospital care. Dr Connolly suggested that the minimum training required could be as little as a one-day course to identify free fluid, tamponade, emboli and cardiac dilation. He highlighted specific benefits to pre-hospital care where noisy scenes may make it considerably easier to ‘look than listen.’ Dr Connolly emphasized how the barriers to widespread ultrasound use in cardiac arrest were artificial and that a proactive approach to the use of ultrasound during cardiac arrest could be associated with better outcomes.

To compliment the academic discussions, a speaker was invited to give an emotional and inspiring personal account of his experience of cardiac arrest. Chris Solomons was working for the Yorkshire Air Ambulance as an Emergency Medical Dispatcher when he suffered chest pain whilst driving to work. He collapsed in the presence of his colleagues who recognised and treated his cardiac arrest. The entire incident was captured on film. In session, Chris described his recollection of the day’s events and his recovery.

Mr. Mark Whitbread, Consultant Paramedic for the London Ambulance Service, ran a session on the future of pre-hospital cardiac arrest management from a paramedic perspective. Cardiac arrest out of hospital differs from that in hospital with neuronal damage and death being most common as opposed to organ failure in hospital. Considering London as an example, cardiac arrest survival has improved from 4.2% (2008) to 31.7% (2012) thus highlighting the improvements in resource allocation, dispatch and triage to appropriate centres for intervention. Despite the progress made in the logistics of a cardiac arrest there are still practices on scene that could be adopted to improve outcome. These include use of pre-hospital ultrasound, cardioversion, external pacing, mechanical CPR and active patient cooling. He closed by suggesting that there is a need for real time feedback, good crew management and comprehensive training to further improve survival.

Dr Barney Scholefield from Birmingham Children’s Hospital, finished the day by presenting on paediatric cardiac arrest. Challenges in paediatric cardiac arrest resuscitation include variation in witnessing of arrests and bystander CPR. Dr Scholefield emphasized the need for rapid bystander CPR. 4% of asystolic arrests in children can gain ROSC up to, and beyond, 30 minutes of CPR. For optimal management Dr Scholefield discussed the need for primary prevention, bystander CPR and intensive paediatric care and monitoring. He closed by highlighting future possibilities in paediatric resuscitation including parental CPR training as part of antenatal care, the role of automated CPR in paediatrics, aggressive management of temperature post arrest and the role of extra corporeal life support.

